# Association of plasma and urine NGAL with acute kidney injury after elective colorectal surgery: A cohort study

**DOI:** 10.1016/j.amsu.2021.01.060

**Published:** 2021-01-22

**Authors:** Nuttha Lumlertgul, Marlies Ostermann, Stuart McCorkell, Jonathan van Dellen, Andrew B. Williams

**Affiliations:** a Department of Critical Care, Guy's & St Thomas' Hospital, London, UK; b Division of Nephrology, Department of Internal Medicine, King Chulalongkorn Memorial Hospital, Bangkok, Thailand; c Excellence Center for Critical Care Nephrology, King Chulalongkorn Memorial Hospital, Bangkok, Thailand; d Research Unit in Critical Care Nephrology, Chulalongkorn University, Bangkok, Thailand; e Department of Anaesthesia, Guy's & St Thomas' Hospital, London, UK; f Department of Colorectal Surgery, Guy's & St Thomas' Hospital, London, UK

**Keywords:** Acute kidney injury, NGAL, Colorectal surgery, Colorectal cancer, Biomarker

## Abstract

**Background:**

Acute kidney injury (AKI) is common in surgical patients. We aimed to investigate the validity of plasma and urine neutrophil gelatinase-associated lipocalin (NGAL) in the detection of AKI and prediction of outcomes in patients undergoing major colorectal surgery.

**Materials and methods:**

This was a pre-specified post-hoc analysis of a randomized controlled trial comparing oesophageal doppler and Lithium dilution cardiac output monitoring in high risk patients undergoing major colorectal surgery as part of an Enhanced Recovery After Surgery protocol in a tertiary care hospital. Plasma and urine samples for NGAL measurement were taken before surgery (T1), immediately after surgery (T2), and on postoperative day 1 (T3). AKI was defined according to the KDIGO criteria.

**Results:**

A total of 89 patients were included of whom 12 (13.5%) developed AKI. Plasma NGAL significantly increased from T1 to T3 in both AKI (p < 0.001) and non-AKI (p = 0.048) patients, while urine NGAL did not change over time. There were no significant differences in plasma and urine NGAL in patients with and without AKI at all time points. Postoperative day 1 urine NGAL concentrations were significantly higher in non-survivors than survivors (41.2 versus 25 ng/mL, p = 0.026). One-year mortality was significantly higher in AKI patients with a raised urine NGAL compared to AKI patients without elevated urine NGAL levels.

**Conclusions:**

Plasma and urine NGAL poorly predicted AKI post-colorectal surgery. Non-survivors had higher urine NGAL results. More research is required to explore the association between NGAL and long-term outcomes.

## Highlights

• Acute kidney injury occurred in 13.5% of patients undergoing major elective colorectal surgery.
• Plasma and urine NGAL poorly predicted AKI after major elective colorectal surgery.
• The association of NGAL and AKI appears to be confounded by malignancy and chronic kidney disease.
• 1-year mortality in AKI patients with a raised urine NGAL was higher than in patients with AKI and a normal urine NGAL value.

## 1 Introduction

Acute kidney injury (AKI) is common in surgical patients and is associated with an increased risk of postoperative complications, longer stay in hospital, and higher mortality [[1], [2], [3], [4], [5], [6]]. There are multiple potential causes [7]. Survivors of AKI are more likely to develop chronic kidney disease (CKD) and have a higher risk of cardiovascular complications and premature mortality [[8], [9], [10], [11]]. Consequently, it is important to diagnose AKI early to initiate appropriate management and potentially prevent AKI-related complications.

The incidence of postoperative AKI after colorectal surgery varies from 3 to 13% [[12], [13], [14], [15], [16]]. Enhanced Recovery After Surgery (ERAS) protocols are multi-modal, multidisciplinary programs aiming to reduce surgical stress and restore physiological functions early. The key components of ERAS protocols are fluid administration guided by goal-directed haemodynamic monitoring, more liberal use of vasopressors, multimodal pain management, early resumption of oral intake, and early mobilisation. These programs are well established in colorectal surgery and have shown to hasten the return of bowel function, reduce postoperative complications and decrease length of hospital stay [[16], [17]]. Whether the implementation of an ERAS program affects the risk of AKI is still controversial [15,16,[18], [19], [20]].

A recent expert consensus meeting recommended using validated AKI biomarkers to identify patient populations who may benefit from early interventions to improve outcomes [21]. Neutrophil gelatinase-associated lipocalin (NGAL) is one of the most extensively studied biomarkers for AKI. It is considered a marker of tubular damage and has shown promising results for early AKI prediction in various settings such as post-cardiac surgery, coronary angiography, kidney and liver transplantation, and critically ill patients [[22], [23], [24]]. Nevertheless, certain conditions such as inflammation, sepsis, and cancer can increase both systemic and urine NGAL concentrations, thus mitigating its robustness as an indicator of renal damage [24].

The utility of plasma NGAL (pNGAL) and urine NGAL (uNGAL) as markers of early postoperative AKI and predictors of outcomes in patients with colorectal malignancy or inflammatory bowel disease is unknown. This study aims to evaluate the association of pNGAL and uNGAL with postoperative AKI in patients undergoing major elective colorectal surgery applying ERAS protocols.

## 2 Methods

### 2.1 Study design

This was a pre-specified post-hoc analysis of a prospective single-centre randomized controlled trial at a tertiary academic hospital in patients undergoing elective colorectal surgery using ERAS protocols from December 2011 until September 2012 (Trial registration: ISRCTN 50251697 and 24020298; Unique identification number: researchregistry6434. Available at https://www.researchregistry.com/browse-the-registry#home/registrationdetails/5ffb1ee4acac01001b8c35b3/). The first series compared intra-operative goal-directed fluid optimization using pulse pressure waveform analysis (LiDCOrapid) with Oesophageal Doppler (ODM, CardioQ-ODM, DeltexMedical, Chichester, UK). In the second phase, oesophageal doppler-guided oxygen delivery index (DO_2_i)-targeted fluid optimization was extended to 16 h after surgery versus standard post-operative care [25,26]. The study was approved by South East London Research Ethics Committee (REC) 2 (Reference No: 11/H0802/9). All patients gave written informed consent and the study was conducted according to the principles of the Declaration of Helsinki. Reporting is consistent with the STROCSS guidelines [27].

### 2.2 Population

We enrolled adults undergoing major high-risk colorectal surgery using ERAS protocols at a tertiary care center in the UK. Criteria for high-risk patients were 1) pre-operative American Society of Anesthesiologists (ASA) physical classification grade III and above, 2) anaerobic threshold <11 ml/min/kg, or 3) undergoing major complex surgery (as per Office of Population, Censuses and Surveys (OPCS) 4.8 Classification of Surgical Operations and Procedures) and planned for overnight intensive recovery (OIR) admission [28]. Exclusion criteria were 1) emergency operations, 2) lack of capacity to consent, 3) pregnancy, 4) contraindication to oesophageal Doppler probe, 5) contraindication to arterial access for LiDCO*rapid* monitoring, 6) previous renal replacement therapy (RRT), or 7) kidney transplant.

### 2.3 Data collection

Baseline characteristics, comorbidities, and perioperative data were collected prospectively from the hospital electronic medical records (EMR) and paper anaesthetic and operation records. Baseline serum creatinine (lowest value within 3 months preceding surgery) and daily serum creatinine up to 3 days post-surgery were recorded. Glomerular filtration rate (GFR) was estimated using the Chronic Kidney Disease Epidemiology Collaboration (CKDEpi) creatinine formula. Chronic kidney disease (CKD) was defined as estimated GFR (eGFR) < 60 mL/min/1.73 m^2^ persisting for 3 months [29]. CardioQ ODM or LiDCO*rapid* were used for haemodynamic cardiac output monitoring to guide fluid management, and additional vasopressor support if required.

### 2.4 Sample collection

Blood and spot urine samples for measurement of pNGAL and uNGAL were prospectively collected pre-operatively after induction of anesthesia and before the start of surgery (T1), immediately after surgery (T2), and on post-operative day 1 (T3). Samples were centrifuged at 3000 g for 4 min and the supernatants were stored at −80 °C until measurement.

### 2.5 NGAL measurement

Plasma NGAL and uNGAL were measured immediately upon study completion using enzyme-linked immunosorbent assay (ELISA) (BioPorto diagnostic A/S, Copenhagen, Denmark). The upper limits were 250 ng/mL for pNGAL and 100 ng/mL for uNGAL as advised by the manufacturer [30].

### 2.6 Outcomes

The primary outcome was new AKI within 72 h after surgery as defined by the serum creatinine (SCr) criteria of the Kidney Disease Improving Global Outcome (KDIGO) classification [31]. Secondary outcomes included post-operative complications according to the Clavien-Dindo classification, re-admission and mortality at 30 days, 6 months, and 1, 5, and 8 years. The data were collated and checked against the patients’ notes following patient discharge. Long-term mortality was collected from the medical electronic patient records.

### 2.7 Statistical analysis

Categorical data were expressed in frequencies and compared using Chi-square or Fisher's exact test, where appropriate. Continuous data were expressed in mean ± standard deviation if normally distributed and compared using independent *t*-test. Non-parametric data were expressed in median (interquartile range, IQR) and compared using Wilcoxon rank-sum test or Kruskall-Wallis test. Univariate logistic regression analysis was performed to assess an association between risk factors and AKI. Multicollinearity was tested, and parameters with p < 0.05 were used for multivariate logistic regression analysis. Differences between pNGAL and uNGAL values with respect to time were analysed using a linear generalized estimating equation (GEE) comparing between AKI and non-AKI groups and changes over time. Spearman's rank correlation was determined between baseline pNGAL and uNGAL and other parameters. To examine the diagnostic performance of pNGAL and uNGAL for the development of AKI, the area under the receiver operating characteristic (AUROC) curve was determined. Unadjusted survival data were plotted as a Kaplan-Meier estimate and compared using the log rank test. A univariate cox-regression analysis was undertaken to assess factors related to time to death. Variables with p < 0.05 were included in the multivariate analyses. A 2-tailed p value < 0.05 was considered statistically significant. All statistical analyses were performed using Stata 14.0 (Statacorp, College Station, Texas, USA).

## 3 Results

### 3.1 Patient characteristics

**Fig. 1:**
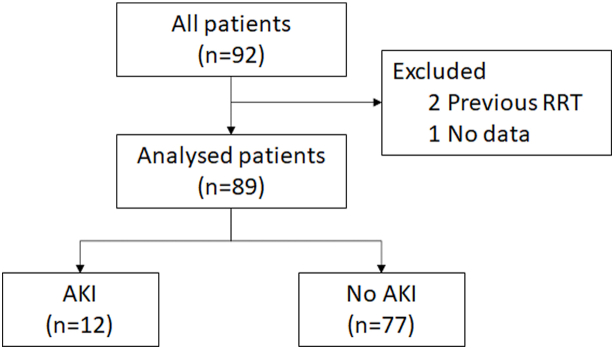
Study flow.

Ninety-two patients participated in the study. After exclusion of two patients who had had RRT prior to surgery and one patient due to missing data, 89 patients were included in the final analysis (51.7% male, mean age 56.5 ± 18.0 years) (Fig. 1). Their main diagnoses were malignancy (49.4%), inflammatory bowel disease (22.5%), and other benign conditions (28.1%) (Table S1).

### 3.2 Incidence of AKI

Twelve patients (13.5%) developed AKI within 72 h after surgery; 9 patients had AKI stage 1, 2 patients stage 2, and 1 patient stage 3, respectively. No patient received RRT. Compared to patients without AKI, AKI patients were characterized by a higher proportion with diabetes, a higher POSSUM physiological score, higher use of combined spinal anesthesia with patient-controlled analgesia (PCA), a lower preoperative haemoglobin, higher red cell transfusion requirement intra-operatively, and a higher C-reactive protein (CRP) (Table 1). After surgery, they were likely to experience more severe complications and had a longer stay in hospital. Six-month and one-year mortality were significantly higher in AKI versus non-AKI patients.

**Table 1:** Baseline characteristics, peri-operative parameters, and outcomes in patients with and without acute kidney injury.

Parameters	All patients	No AKI (n = 77)	AKI (n = 12)	*P* value
Baseline characteristics				
Male sex	46 (51.7)	37 (48.1)	9 (75)	0.121*
Age (years)	56.5 ± 18.0	55.4 ± 18.0	63.2 ± 16.8	0.166
Weight (kg)	77.9 ± 17.0	78.1 ± 17.5	76.7 ± 14.1	0.781
BMI (kg/m^2^)	27.0 ± 5.6	27.2 ± 5.8	26.3 ± 4.1	0.614
COPD	10 (11.2)	8 (10.4)	2 (16.7)	0.619*
Diabetes	11 (12.4)	7 (9.1)	4 (33.3)	0.038*
CVA	4 (4.5)	4 (5.2)	0	1.000*
Hypertension	19 (21.4)	17 (22.1)	2 (16.7)	1.000*
Ischemic heart disease	3 (3.4)	3 (3.9)	0	1.000*
Smoker	19 (21.8)	17 (22.7)	2 (16.7)	1.000*
Haemoglobin (g/dL)	12.9 ± 1.9	13.1 ± 1.8	11.6 ± 2.1	0.007
Pre-morbid anaemia	30 (34.1)	22 (28.9)	8 (66.7)	0.019*
Baseline Cr (μmol/L)	73 (60, 87)	71 (61, 82)	84 (66.5, 98.5)	0.065
Baseline GFR (mL/min/1.73m^2^)	91.9 (72.9, 108.3)	92.5 (75.0, 108.7)	74.3 (68.0, 99.5)	0.120
CKD	10 (11.2)	8 (10.4)	2 (16.7)	0.619*
**Perioperative factors**				
Complexity				
- CMO	41 (46.1)	35 (45.5)	6 (60)	0.769
- MAJ	48 (53.9)	42 (54.5)	6 (50)	
Diagnosis				
- Malignancy	44 (49.4)	36 (46.8)	8 (66.7)	0.518*
- IBD	20 (22.5)	18 (23.4)	2 (16.7)	
- Other	25 (28.1)	23 (29.9)	2 (16.7)	
Type of operation				
- Open	74 (83.2)	63 (81.8)	11 (91.7)	0.682*
- Laparoscopy	15 (16.8)	14 (18.2)	1 (8.3)	
New stoma	41 (46.1)	37 (48.1)	4 (33.3)	0.373*
POSSUM Physiological score	16 (13.5, 18)	15 (13, 17)	18 (14, 24.5)	0.019
POSSUM Operative severity score	17.5 (12,22.5)	14.5 (11,20.5)	17.5 (12,22.5)	0.284
POSSUM Total score	32 (27,38.5)	32 (26.5,37)	38.5 (32.5,43)	0.009
ASA classification				
- 1	13 (14.6)	11 (14.3)	2 (16.7)	0.386*
- 2	58 (65.2)	52 (67.5)	6 (50)	
- 3	18 (20.2)	14 (18.2)	4 (33.3)	
Analgesia				
- PCA	26 (29.2)	25 (32.5)	1 (8.3)	0.058
- Spinal/epidural	37 (41.6)	33 (42.9)	4 (33.3)	
- Combined	26 (29.2)	19 (24.7)	7 (58.3)	
Start surgery stroke volume (ml)	79 (67, 92)	80 (67, 92)	75 (67, 93.5)	0.532
Start surgery cardiac output (L/min)	5.5 (4.9, 7.2)	5.5 (4.9, 6.8)	5.6 (4.9, 8.5)	0.601
Start surgery cardiac index (L/min/m^2^)	3.0 (2.6, 3.7)	3.0 (2.6, 3.7)	3.0 (2.4, 4.2)	0.829
Start surgery FTC (millisec)	375 (336, 401)	374 (338, 397)	376.5 (325, 415.5)	0.885
Start surgery haemoglobin (g/dL)	11.4 (10.2, 12.7)	11.6 (10.3, 12.8)	10.4 (8.5, 11.6)	0.037
Start surgery lactate (mmol/L)	1.8 (1.4, 2.3)	1.7 (1.3, 2.3)	2.1 (1.7, 2.8)	0.129
Start surgery oxygen saturation (%)	99.7 (99.6, 99.8)	99.7 (99.6, 99.8)	99.7 (99.6, 99.8)	0.782
Baseline DO_2_i (mL/min/m^2^)	463.7 (381.9, 554.1)	464.7 (383.7, 553.4)	4637 (308.3, 558)	0.683
Intra-operative blood transfusion units	0 (0, 0)	0 (0, 0)	0 (0, 2)	0.014
Intra-operative catecholamine therapy	55 (61.8)	48 (62.3)	7 (58.3)	0.791
Operative time (min)	174 (127, 238)	163.5 (127, 238)	226.5 (141, 242)	0.366
Intra-operative fluid (mL)				
- Crystalloids	2000 (1100, 3000)	2000 (1000,3000)	2000 (1400,3000)	0.651
- Colloids	1000 (500, 2000)	1000 (500, 2000)	500 (500, 2000)	0.491
- Blood products	0 (0, 440)	0 (0, 0)	0 (0, 560)	0.508
- Total	3562.5 (2000, 5000)	3625 (2500, 5000)	3500 (1900, 4500)	0.771
**Outcomes**				
CRP**	138 (92, 237)	131 (59, 234)	205 (151, 289)	0.024
Highest post-operative SCr (μmol/L)^#^	80 (61, 100)	74 (59, 89)	130 (103, 222.5)	<0.001
Clavien-Dindo classification				
0	34 (38.2)	34 (44.2)	0	0.003*
I - II	52 (58.4)	40 (52)	12 (100)	0.001*
III - IV	15 (16.9)	8 (10.4)	7 (58.3)	<0.001
OIR length of stay (hours)	18.5 (16.8, 21.5)	18.5 (16.4, 21.3)	18.5 (17.5, 22.5)	0.694
Hospital length of stay (days)	10 (6, 14)	9 (6, 12)	17 (8.5, 24)	0.046
30-day readmission (%)	13 (14.6)	9 (11.7)	4 (33.3)	0.070*
30-day mortality (%)	1 (1.1)	0	1 (8.3)	0.135*
6-mo mortality (%)	3 (3.4)	1 (1.3)	2 (16.7)	0.047*
1-year mortality (%)	6 (6.7)	2 (2.6)	4 (33.3)	0.003
5-year mortality (%)	20 (22.5)	15 (19.5)	5 (41.7)	0.087
8-year mortality (%)	24 (27.0)	19 (24.7)	5 (41.7)	0.217

Abbreviations: AKI, acute kidney injury; BMI, body mass index; COPD, chronic obstructive pulmonary disease; CVA, cerebrovascular accident; Cr, creatinine; GFR, glomerular filtration rate; CKD, chronic kidney disease; CMO, complex major operation; MAJ, major surgery; POSSUM, Physiological and Operative Severity Score for the enUmeration of Mortality and Morbidity; IBD, inflammatory bowel disease; ASA, American Society of Anaesthesiology; PCA, patient-controlled analgesia; FTC, corrected flow time; DO_2_i, oxygen delivery index; CRP, c-reactive protein; OIR, overnight intensive recovery.

* Fisher's exact test.

** Immediately after surgery.

^#^ Highest creatinine within 72 h after surgery.

### 3.3 Trajectories of NGAL

**Fig. 2:**
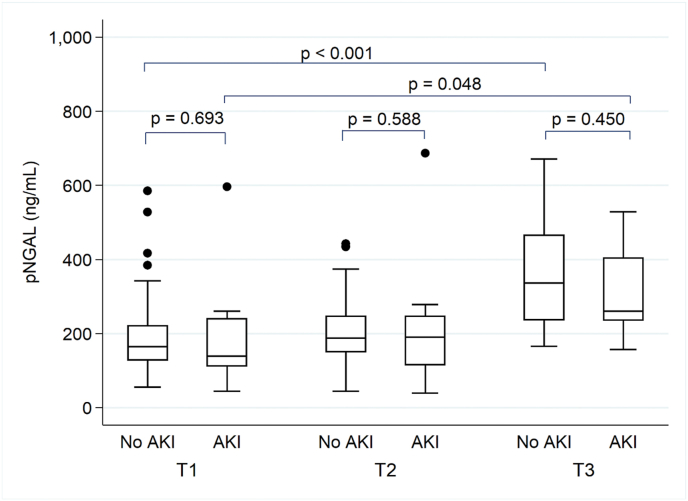
Plasma neutrophil gelatinase-associated lipocalin (pNGAL) before surgery (T1), immediately after surgery (T2), and post-operative day 1 (T3) between AKI and non-AKI patients. Box plots indicate the median. Error bars indicate 25th and 75th percentile.

**Fig. 3:**
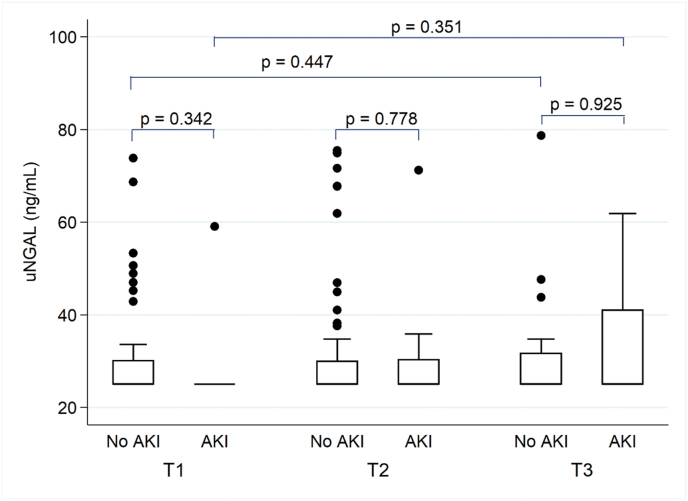
Urine neutrophil gelatinase-associated (uNGAL) before surgery (T1), immediately after surgery (T2), and postoperative day 1 (T3) between AKI and non-AKI patients. Box plots indicate the median. Error bars indicate 25th and 75th percentile.

There was a significant increase in pNGAL concentrations from T1 to T3 in non-AKI (p = 0.048) and AKI patients (P < 0.001), but there was no significant difference between both groups (p = 0.884) (Table 2, Fig. 2). Serial uNGAL values did not significantly change and there was no difference between patients with and without AKI (p = 0.351). (Fig. 3).

**Table 2:** **Trajectories of pNGAL and uNGAL**.

Outcome	No AKI				AKI				p between groups^a^
	T1 (n = 75)	T2 (n = 75)	T3 (n = 26)	p within groups^a^	T1 (n = 11)	T2 (n = 12)	T3 (n = 9)	p within groups^a^	
pNGAL [ng/ml]	164.6 (128, 220.9)	188.1 (149.9, 246.5)	336.6 (236.4, 465.6)	0.048	138.9 (112.1, 240)	190.6 (115.5, 247.1)	260.1 (236, 403.9)	<0.001	0.884
	**T1 (n = 74)**	**T2 (n = 75)**	**T3 (n = 28)**		**T1 (n = 11)**	**T2 (n = 12)**	**T3 (n = 9)**		
uNGAL [ng/ml]	25 (25, 32.6)	25.7 (25, 67.8)	27.3 (25, 45.7)	0.447	25 (25, 25)	27.7 (25, 129.5)	25 (25, 48.4)	0.699	0.351

Abbreviations: AKI, acute kidney injury; pNGAL, plasma neutrophil gelatinase-associated lipocalin; uNGAL, urine neutrophil gelatinase-associated lipocalin.

T1 = Before surgery; T2 = Immediately after surgery; T3 = Post-operative day 1.

a All analyses were performed by generalized estimated equations.

### 3.4 Risk factors for AKI

Multivariate logistic regression identified log10CRP and intra-operative blood transfusion as independent predictors for AKI (Table 3). Neither pNGAL nor uNGAL was associated with AKI development. The AUROC curve of pNGAL for predicting post-operative AKI ranged from 0.43 (95% CI 0.17–0.69) (T3) to 0.46 (0.19–0.74) (T2). The maximal AUROC curve of uNGAL was 0.54 (0.31–0.77) at T2 (Fig. S1 and Fig. S2).

**Table 3:** Univariate and multivariate logistic regression for AKI prediction.

Parameters	Crude odds ratio (95% CI)	p value	Adjusted odds ratio (95% CI)	p value
Diabetes	5 (1.20, 20.89)	0.027	3.87 (0.52, 28.68)	0.185
Pre-operative haemoglobin	0.65 (0.47, 0.91)	0.012	0.64 (0.36, 1.14)	0.130
POSSUM physiological score	1.17 (1.05, 1.32)	0.007	0.98 (0.83, 1.22)	0.829
POSSUM total score	1.11 (1.03, 1.20)	0.010		
Analgesia				
• PCA only	1	Reference	1	Reference
• Epidural/spinal anaethesia	3.03 (0.32, 28.81)	0.335	9.26 (0.55, 155.62)	0.122
• Combined	9.21 (1.04, 81.36)	0.046	10.04 (0.69, 145.27)	0.091
Log10 CRP	3.73 (1.10, 12.62)	0.034	6.05 (1.27, 28.89)	0.024
Intra-op blood transfusion units	1.45 (1.04, 2.02)	0.028	1.59 (1.04, 2.41)	0.031
Log10 pNGAL at baseline	0.77 (0.22, 2.69)	0.681		
Log10 uNGAL at baseline	2.80 (0.94, 8.38)	0.067		

Abbreviations: POSSUM, Physiological and Operative Severity Score for the enUmeration of Mortality and Morbidity; PCA, patient-controlled analgesia; CRP, c-reactive protein; pNGAL, plasma neutrophil gelatinase-associated lipocalin; uNGAL, urine neutrophil gelatinase-associated lipocalin.

### 3.5 Association of NGAL and underlying disease

The median concentrations of plasma and urine NGAL were similar in malignancy, inflammatory bowel diseases, and other benign diagnoses. Plasma NGAL was higher in CKD patients and in more advanced colorectal cancer, whereas uNGAL was significantly higher in metastatic cancer (M1) (Table 4; Fig. S3). Spearman's rank correlation demonstrated baseline pNGAL to be positively associated with tumour staging (p = 0.017) and uNGAL (p = 0.006). (Table S2; Fig. S4).

**Table 4:** Relationship of baseline pNGAL and uNGAL and clinical characteristics.

Characteristics	Number of patients	pNGAL [ng/ml]	p value	uNGAL [ng/ml]	P value
Diagnosis^a^					
• Malignancy	44	161.6 (117.6, 241.1)	0.744	25 (25, 42.9)	0.868
• IBD	20	163.1 (128.5, 202.4)		25 (25, 31.8)	
• Others	25	176.3 (131.9, 255.7)		25 (25, 28)	
Diabetes^b^					
• No	78	161.3 (127.9, 215.9)	0.549	25 (25, 31.8)	0.176
• Yes	11	196.2 (115.6, 220.9)		30.1 (25, 59.1)	
CKD ^b^					
• No	79	161.3 (120.4, 203.3)	0.035	25 (25, 31.9)	0.547
• Yes	10	277.4 (140.2, 310.9)		25 (25, 42.9)	
Type of operation ^b^					
• Open	74	164.6 (127.8, 240)	0.484	25 (25, 32.6)	0.627
• Laparoscopy	15	139.3 (117.6, 187.6)		25 (25, 48.9)	
Malignancy staging	44				
T stage^a^					
• T1/T2	12	116.6 (102.7, 154.3)	0.019	25 (25, 28.1)	0.489
• T3	25	176.9 (138.4, 313.4)		25 (25, 42.9)	
• T4	7	177.6 (123.2, 202.9)		25.8 (25, 184.8)	
N stage^b^					
• N0	26	138.4 (115.6, 176.9)	0.056	25 (25, 45.2)	0.932
• N1/2	18	178.5 (146.5, 308.9)		25 (25, 33.3)	
M stage^b^					
• M0	40	146.5 (117.6, 260.1)	0.587	25 (25, 33)	0.007
• M1	4	195.8 (145.0, 227.1)		56.2 (39.9161.6)	
IBD^b^					
• UC	10	153.1 (137.3, 176.0)	0.722	25 (25, 25)	0.214
• Crohn's disease	10	178.9 (117.1, 203.6)		27.6 (25, 33.6)	

Abbreviations: IBD, inflammatory bowel diseases; CKD, chronic kidney disease; T, tumour; N, nodes; M, metastases; UC, ulcerative colitis; pNGAL, plasma neutrophil gelatinase-associated lipocalin; uNGAL, urine neutrophil gelatinase-associated lipocalin.

a- Kruskal wallis test; b-Wilcoxon rank-sum test.

### 3.6 NGAL and long-term outcome

Twenty-four patients died within 8 years after surgery (27.0%), of whom 20.8% had postoperative AKI compared with 10.8% among survivors (Table S3). Causes of death were cancer progression (n = 10), sepsis (n = 3), and stroke (n = 2); the cause of death of the remaining 9 patients could not be determined. There was a trend towards higher 8-year mortality in patients with more severe AKI. In non-survivors, pNGAL (T1 and T2) and immediate postoperative uNGAL (T2) were marginally higher, and postoperative day 1 uNGAL was significantly higher in non-survivors than survivors (41.2 ng/mL [IQR 27.7, 101.4] *versus* 25 ng/mL [IQR 25, 31.5]), p = 0.026.

Univariate cox-regression analysis showed that pNGAL (T1 and T2) and uNGAL after surgery (T2 and T3) were associated with increased mortality (Table 5). Multivariate cox-regression analysis revealed that only POSSUM physiological score was associated with 8-year mortality.

**Table 5:** Univariate and multivariate cox-regression analysis and 8-year mortality.

Parameters	Crude HR (95% CI)	p value	Adjusted HR (95% CI)	p value
Age^a^	1.06 (1.03, 1.09)	<0.001	–	
Diabetes	3.01 (1.19, 7.59)	0.019	1.04 (0.22, 4.90)	0.956
Malignancy	2.80 (1.16, 6.77)	0.022	1.37 (0.43, 4.39)	0.597
T stage				
• T1/T2	1	Reference	–	
• T3	1.81 (0.50, 6.58)	0.368		
• T4	4.25 (0.95, 19.09)	0.059		
N stage				
• N0	1	Reference	–	
• N1	1.43 (0.51, 4.01)	0.501		
• N2	1.91 (0.41, 8.88)	0.409		
Metastases	9.35 (2.58, 33.89)	0.001	–	
IBD	0.13 (0.02, 0.96)	0.045	0.30 (0.03, 2.92)	0.299
Pre-operative haemoglobin (per each 1 g/dL)^a^	0.71 (0.58, 0.88)	0.001	–	
Pre-operative anaemia^a^	3.10 (1.36, 7.07)	0.007	–	
Hypertension	4.38 (1.96, 9.82)	<0.001	1.44 (0.44, 4.73)	0.546
CKD	3.45 (1.43, 8.34)	0.006	0.92 (0.20, 4.17)	0.916
Previous CVA	4.31 (1.27, 14.68)	0.019	2.14 (0.30,15.10)	0.445
POSSUM Physiological score	1.21 (1.12, 1.30)	<0.001	1.19 (1.04, 1.36)	0.010
POSSUM total score^b^	1.09 (1.04, 1.14)	<0.001	–	
Clavien-Dindo classification III-IV	4.34 (1.89, 9.97)	0.001	2.87 (0.63,13.07)	0.173
Highest Cr (per each μmol/L)	1.01 (1.01, 1.02)	<0.001	1.00 (0.99, 1.01)	0.931
AKI				
- No AKI	1	Reference	–	
- AKI stage 1	1.53 (0.45, 5.16)	0.495		
- AKI stage 2	3.49 (0.47, 26.07)	0.224		
- AKI stage 3	18.55 (2.15, 160.17)	0.008		
Log10 pNGAL (T1)	2.31 (1.03, 5.19)	0.042	0.62 (0.12, 3.13)	0.566
Log10 pNGAL (T2)	3.21 (1.20, 8.56)	0.020	1.54 (0.30, 7.93)	0.604
Log10 pNGAL (T3)	1.74 (0.42, 7.12)	0.442	–	
Log10 uNGAL (T1)	1.40 (0.90, 2.19)	0.134	–	
Log10 uNGAL (T2)	1.46 (1.02, 2.10)	0.040	1.50 (0.86, 2.61)	0.151
Log10 uNGAL (T3)^c^	1.57 (1.01, 2.44)	0.047	–	

Abbreviations: T, tumour; N, nodes; IBD, inflammatory bowel diseases; CKD, chronic kidney disease; CVA, cerebrovascular accident; POSSUM, Physiological and Operative Severity Score for the enUmeration of Mortality and Morbidity; CRP, c-reactive protein; Cr, serum creatinine; AKI, acute kidney injury; pNGAL, plasma neutrophil gelatinase-associated lipocalin; uNGAL, urine neutrophil gelatinase-associated lipocalin.

a Not included in multivariable cox regression analysis because these factors comprise POSSUM physiological score.

b Not included in multivariable cox regression analysis because POSSUM total score showed mulcollinearity with POSSUM physiological score.

c Not included in multivariable cox regression analysis because there were 32 observations.

### 3.7 Sub-group analyses

Using a cut-off value of 250 ng/mL for pNGAL and 100 ng/mL for uNGAL (NGAL+) and presence of AKI (Cr+), patients were further categorized into 4 categories; NGAL-/Cr-, NGAL+/Cr-, NGAL-/Cr+, NGAL+/Cr+ (Table S4). Of all patients, 78.7% and 65.2% patients were classified as pNGAL+/Cr- and uNGAL+/Cr-. One-year mortality was highest in AKI patients with a raised uNGAL concentration (uNGAL+/Cr+) but there was no statistically significant difference in 8-year mortality between AKI patients with and without raised NGAL concentrations. The crude HR for pNGAL+/Cr+ and uNGAL+/Cr + for 8-year mortality are 4.30 [95% confidence interval (CI) 0.48–38.47; p = 0.192) and 5.53 (95% CI 1.38–22.16; p = 0.016) compared to other groups, respectively (Fig. S5 and Fig. S6).

## 4 Discussion

Our study demonstrated a 13.5% incidence of AKI post-elective major colorectal surgery. Most patients had AKI stage 1, and none required RRT. There was an increase in pNGAL and uNGAL between baseline and the immediate postoperative period, but no significant difference between AKI and non-AKI patients. Both plasma and urine NGAL concentrations were higher in non-survivors, and AKI patients with uNGAL positivity demonstrated the worst long-term prognosis.

NGAL has been studied extensively post cardiac surgery [32]. Less is known about its role in major non-cardiac surgery; in addition, the results are conflicting [[33], [34], [35], [36], [37], [38], [39], [40], [41], [42], [43]]. A previous study in 22 postoperative oncological patients, including 15 patients with gastrointestinal tract malignancy, showed that uNGAL was an independent predictor of AKI. None of the patients had CKD, and the association with tumour staging was not explored [43].

NGAL is not organ specific. The employed NGAL assay used in our study measures a monomeric form of NGAL which is predominantly synthesized and released from kidney tubules, but small amounts are also released from inflammatory cells [44]. As a result, NGAL concentrations might have been influenced in patients with pre-existing malignancy or systemic inflammatory conditions. In our patient cohort, pNGAL increased significantly over time, which might suggest tissue injury or direct renal insult intraoperatively and post-surgery but there was no association with development of AKI. However, confounding may have occurred, for example, by malignant disease or the presence of CKD.

Our results suggest an association of NGAL and malignant disease. Pathologically, NGAL expression in colorectal tissue has been shown to correlate with advanced TNM stage, depth of tumor invasion, presence of lymph node metastases, and decreased disease-free survival [45,46]. Several studies have shown that serum NGAL concentrations were higher in cancer patients compared with benign diagnoses or healthy controls, and that serum NGAL correlated with T staging, maximal tumor size, and lymph node involvement [[47], [48], [49], [50]]. Previous studies also suggested that serum NGAL was able to distinguish active inflammatory bowel disease from inactive disease [51,52]. In our study, about one-fourth of our patients with inflammatory bowel disease had similar NGAL concentrations to those with malignancy.

Interestingly, non-survivors at 8 years were characterized by higher baseline and postoperative pNGAL concentrations and significantly higher postoperative uNGAL (T3) values compared to survivors. pNGAL was shown an independent predictor for long-term mortality in cancer settings [46]. Our study showed that AKI patients with uNGAL positivity (NGAL+/Cr+) had worse 1-year prognosis than those with AKI and normal uNGAL concentrations (NGAL-/Cr+). This could possibly be explained by association between uNGAL and metastases. Whether NGAL can predict long-term prognosis independent of cancer progression and AKI needs to be determined in future studies.

This is the largest study to date which explored the role of NGAL after major colorectal surgery. However, it is important to acknowledge some limitations. This was a single-center study with limited sample size and small event rates, and a type II error cannot be excluded. Urine output was not used for AKI diagnosis and staging, which might have underestimated the true incidence of AKI. We did not collect any long-term creatinine results and cannot comment on the association between NGAL and development of CKD. Finally, causes of death could not be determined for all patients; therefore, we cannot associate NGAL with long-term mortality independent of cancer progression. Nevertheless, our study is the first to examine the utility of both plasma and urine NGAL for AKI prediction after colorectal surgery in the era of ERAS protocols. Both, pNGAL and uNGAL were measured at three time points to compare changes within and between groups. All patients underwent rigorous haemodynamic monitoring before and after surgery.

In summary, in patients undergoing colorectal surgery under ERAS protocol, the impact of underlying malignant disease and chronic comorbidities precludes NGAL as a reliable biomarker for AKI prediction.

## Ethical approval

The study has ethical approval from the Research & Ethics Committee South East London 2 (Reference 11/H0802/9). Substantial amendment (version 1.2) of addition of urinary NGAL assays, with paired serum NGAL & CRP assays, to increase sensitivity of acute kidney injury has also been approved.

## Funding

This study was funded by a New Services and Innovation Grant awarded by the Guy's & St Thomas' Charities. A full-time research assistant was funded jointly with contribution by the medical device manufacturer, Deltex Medical (Chichester, UK).

## Author contribution

MO, JvD and AW designed the study. JvD and NL collected the data and performed the data analysis. All authors contributed to the interpretation of the results. NL wrote the first draft of the manuscript. MO, AW and JvD edited the manuscript. All authors met criteria for authorship and approved the final manuscript.

## Trial registration

ISRCTN 50251697 and ISRCTN 24020298, UIN researchregistry6434.

## Guarantor

Marlies Ostermann accept full responsibility for the work and/or the conduct of the study, had access to the data, and controlled the decision to publish.

## Consent

Informed consent was obtained from all individual participants included in the study.

## Provenance and peer review

Not commissioned, externally peer-reviewed.

## Declaration of competing interest

The authors declare that they have no conflict of interest.
