# Supplementary material for: Association of plasma and urine NGAL with acute kidney injury after elective colorectal surgery: A cohort study
Source: Ann Med Surg (Lond). 2021 Jan 22;62:315–22. doi: 10.1016/j.amsu.2021.01.060 (PMC7847815; doi:10.1016/j.amsu.2021.01.060)
Supplement: Multimedia component 2 [file mmc2.docx]

**Supplementary material**

**Supplementary Table S1** **Types of surgery categorized by complexity**

| **Complex major surgery** | **Major surgery** |
| --- | --- |
| Abdominoperineal excision of rectum | Reversal ileostomy/colostomy |
| Proctocolectomy | Stoma formation / stoma closure |
| Pouch surgery | Hemicolectomy |
| Low anterior resection or total mesorectal excision (anastomosis within 7cm of anal verge) | Subtotal colectomy with / without ileorectal anastomosis |
|  | Hartmanns reversal |
|  | High anterior resection (anastomosis > 7 cm from anal verge) |
|  | Ileocaecal resection or right hemicolectomy |

**Supplementary table S2** **Spearman’s correlation coefficients between baseline plasma and urine neutrophil gelatinase-associated lipocalin and clinical parameters**

| **Correlation** | **pNGAL (T1)** | **P value** | **uNGAL (T1)** | **P value** |
| --- | --- | --- | --- | --- |
| Age | 0.19 | 0.080 | 0.10 | 0.373 |
| Weight | 0.08 | 0.441 | -0.07 | 0.484 |
| Haemoglobin (g/dL) | -0.02 | 0.847 | -0.22 | 0.043 |
| CRP (mg/L) | 0.05 | 0.624 | -0.007 | 0.950 |
| POSSUM physiological score | 0.07 | 0.499 | 0.17 | 0.134 |
| POSSUM total score | 0.17 | 0.124 | 0.07 | 0.528 |
| Highest Cr (µmol/L) | 0.14 | 0.189 | -0.05 | 0.642 |
| pNGAL (T1) | - | - | 0.30 | 0.006 |
| T stage | 0.36 | 0.017 | 0.18 | 0.239 |
| N stage | 0.30 | 0.052 | 0.01 | 0.940 |
| Start surgery SV (mL) | 0.17 | 0.126 | 0.06 | 0.599 |
| Start surgery CO (L/min) | 0.19 | 0.086 | 0.12 | 0.282 |
| Start surgery CI (L/min/m^2^) | 0.12 | 0.290 | 0.13 | 0.245 |
| Start surgery FTC (millisec) | 0.11 | 0.315 | 0.07 | 0.498 |
| Start surgery lactate (mmol/L) | -0.29 | **0.018** | 0.12 | 0.297 |
| Start surgery SO_2_ (%) | -0.26 | **0.011** | 0.07 | 0.538 |
| Start surgery DO_2_i (mL/min/m^2^) | 0.17 | 0.121 | 0.06 | 0.590 |

Abbreviations: CRP, c-reactive protein; POSSUM, Physiological and Operative Severity Score for the enUmeration of Mortality and Morbidity; Cr, creatinine; T, tumour; N, nodes; SV, stroke volume; CO, cardiac output; CI, cardiac index; FTC, corrected flow time; SO_2_, oxygen saturation; DO_2_i, delivered oxygen

**Supplementary table S3** **Comparison of baseline characteristics, peri-operative parameters, and outcomes between 8-year survivors and non-survivors**

| **Parameters** | **All patients** | **Survivors**  **(n=65)** | **Non-survivors (n=24)** | **P value** |
| --- | --- | --- | --- | --- |
| Male | 46 (51.7) | 32 (49.2) | 14 (58.3) | 0.446 |
| Age (years) | 56.5 ± 18.0 | 51.1 ± 16.8 | 70.9 ± 12.6 | <0.001 |
| Weight (kg) | 77.9 ±17.0 | 77.9 ± 18.0 | 78.0 ± 14.4 | 0.989 |
| BMI (kg/m^2^) | 27.0 ± 5.6 | 27.1 ± 6.1 | 26.9 ± 3.9 | 0.889 |
| COPD | 10 (11.2) | 7 (10.8) | 3 (12.5) | 1.00* |
| Diabetes | 11 (12.4) | 5 (7.7) | 6 (25.0) | 0.028 |
| CVA | 4 (4.5) | 1 (1.5) | 3 (12.5) | 0.058* |
| Hypertension | 19 (21.4) | 7 (10.8) | 12 (50.0) | <0.001 |
| Ischemic heart disease | 3 (3.4) | 2 (3.1) | 1 (4.2) | 1.000* |
| Smoker | 19 (21.8) | 18 (28.6) | 1 (4.2) | 0.018* |
| Haemoglobin (g/dL) | 12.9 ± 1.9 | 13.3 ± 1.7 | 11.9 ± 2.2 | 0.003 |
| Pre-morbid anaemia | 30 (34.1) | 17 (26.2) | 13 (56.5) | 0.008 |
| Complexity   - CMO - MAJ | 41 (46.1)  48 (53.9) | 29 (44.6)  36 (55.4) | 12 (50.0)  12 (50.0) | 0.651 |
| Diagnosis   - Malignancy - IBD - Other | 44 (49.4)  20 (22.5)  25 (28.1) | 27 (41.5)  19 (29.2)  19 (29.2) | 17 (70.8)  1 (4.2)  6 (25.0) | 0.014  0.011*  0.694 |
| Type of operation   - Open - Laparoscopy | 74 (83.2)  15 (16.9) | 53 (80.3)  13 (19.7) | 21 (91.3)  2 (8.7) | 0.338* |
| New stoma | 41 (46.1) | 30 (46.2) | 11 (45.8) | 0.979 |
| POSSUM Physiological score | 16 (13.5, 18) | 14 (13, 17) | 19 (16, 25) | <0.001 |
| POSSUM Operative severity score | 15 (11.5, 21) | 14 (11, 20) | 18 (12, 23) | 0.231 |
| POSSUM Total score | 32 (27, 38.5) | 31 (25, 36) | 38 (33, 43) | 0.0008 |
| ASA classification   - 1 - 2 - 3 | 13 (14.6)  58 (65.2)  18 (20.2) | 13 (20.0)  46 (70.8)  6 (9.2) | 0  12 (50.0)  12 (50.0) | <0.001 |
| Analgesia   - PCA - Spinal/epidural - Combined | 26 (29.2)  37 (41.6)  26 (29.2) | 20 (30.8)  25 (38.5)  20 (30.8) | 6 (25.0)  12 (50.0)  6 (25.0) | 0.619 |
| Baseline Cr (μmol/L) | 73 (60, 87) | 66 (57, 80) | 84.5 (72.5, 95.5) | 0.0006 |
| Baseline GFR (mL/min/1.73m^2^) | 91.9 (72.9, 108.3) | 100.3 (86.0, 113.1) | 74.4 (55.9, 89.7) | <0.001 |
| CKD | 10 (11.2) | 3 (4.6) | 7 (29.2) | 0.003* |
| Peak Cr (μmol/L) | 80 (61, 100) | 75 (59, 89) | 94 (68.5, 117) | 0.004 |
| Start surgery stroke volume (ml) | 79 (67, 92) | 79 (67, 88) | 81.5 (67.5, 103) | 0.267 |
| Start surgery cardiac output (L/min) | 5.5 (4.9, 7.2) | 5.5 (4.8, 6.5) | 5.9 (5.1, 7.6) | 0.240 |
| Start surgery cardiac index (L/min/m^2^) | 3.0 (2.6, 3.7) | 2.9 (2.6, 3.5) | 3.3 (2.5, 3.9) | 0.300 |
| Start surgery FTC (millisec) | 375 (336, 401) | 373 (338, 395) | 378 (333, 407.5) | 0.654 |
| Start surgery haemoglobin (g/dL) | 11.4 (10.2, 12.7) | 11.8 (10.5, 12.9) | 10.3 (9.1, 12.1) | 0.017 |
| Start surgery lactate (mmol/L) | 1.8 (1.4, 2.3) | 1.7 (1.3, 2.4) | 1.9 (1.4, 2.3) | 0.805 |
| Start surgery SO_2_ (%) | 99.7 (99.6, 99.8) | 99.7 (99.5, 99.8) | 99.7 (99.6, 99.8) | 0.706 |
| Start surgery DO_2_i (mL/min/m^2^) | 463.7  (381.9, 554.1) | 466.6  (389.4, 566.1) | 451.6  (340, 551.4) | 0.547 |
| CRP (mg/L) | 138 (92, 237) | 139 (92, 229) | 136 (96, 263) | 0.585 |
| Intra-operative catecholamine therapy | 55 (61.8) | 38 (58.5) | 17 (70.8) | 0.286 |
| Operative time (min) | 174 (127, 238) | 170 (132, 225) | 229 (107, 353) | 0.299 |
| Intra-operative fluid (mL)   - Crystalloids - Colloids - Blood products - Total | 2000 (1100, 3000)  1000 (500, 2000)  0 (0, 440)  3563 (2000, 5000) | 2000 (1300, 3000)  1000 (500, 2000)  0 (0, 0)  3670 (2500, 4800) | 2000 (1100, 3000)  812.5 (500, 2125)  0 (0, 560)  3518 (1730, 5375) | 0.685  0.701  0.461  0.650 |
| End of surgery stroke volume (ml) | 98 (82, 115) | 94 (75, 112) | 102 (90, 115) | 0.238 |
| End of surgery cardiac output (L/min) | 7.6 (6.2, 8.6) | 7.6 (6, 8.4) | 7.7 (6.3, 9.3) | 0.554 |
| End of surgery cardiac index (L/min/m^2^) | 4 (3.3, 4.8) | 4.1 (3.3, 4.6) | 4.2 (3.3, 5.0) | 0.651 |
| End of surgery FTC (millisec) | 374 (352, 401) | 379 (358, 403) | 361.5 (352, 397.5) | 0.221 |
| End of surgery haemoglobin (g/dL) | 10.4 (9.6, 11.4) | 10.5 (9.7, 12.1) | 9.7 (9.6, 11) | 0.372 |
| End of surgery lactate (mmol/L) | 2.4 (1.7, 3.2) | 2.5 (1.8, 3.4) | 2.2 (1.3, 3.0) | 0.275 |
| End of surgery SO_2_ (%) | 99.7 (99.5, 99.7) | 99.7 (99.6, 99.8) | 99.6 (99.5, 99.7) | 0.186 |
| End of surgery DO_2_i (mL/min/m^2^) | 508 (425, 619.7) | 506 (415.4, 642.4) | 537 (439.5, 581.4) | 0.487 |
| pNGAL (T1) (ng/mL) | 164 (128, 221) | 159 (117, 204) | 179 (139, 260) | 0.076 |
| pNGAL (T2) (ng/mL) | 188.1  (145, 246.5) | 184.5  (132.4, 241.5) | 217.1  (165.7, 278.8) | 0.071 |
| pNGAL (T3) (ng/mL) | 330.9  (236, 465.6) | 332.7  (224.7, 463.0) | 276.3  (254.7, 607.2) | 0.570 |
| uNGAL (T1) (ng/mL) | 25 (25, 32.6) | 25 (25, 30.5) | 25 (25, 42.9) | 0.195 |
| uNGAL (T2) (ng/mL) | 25.7 (25, 71.2) | 25 (25, 53.4) | 34.3 (25, 187.7) | 0.071 |
| uNGAL (T3) (ng/mL) | 25.7 (25, 47.6) | 25 (25, 31.5) | 41.2 (27.7, 101.4) | 0.026 |
| Clavien-Dindo classification |  |  |  |  |
| 0 | 34 (38.2) | 28 (43.1) | 6 (25.0) | 0.119 |
| I-II | 52 (58.4) | 35 (53.9) | 17 (70.8) | 0.149 |
| III-IV | 15 (16.7) | 6 (9.2) | 9 (37.5) | 0.002 |
| OIR duration (hours) | 18.5 (16.8, 21.5) | 18.3 (16.8,17) | 19.8 (17,22) | 0.320 |
| Hospital length of stay (days) | 10 (6, 14) | 8 (6, 12) | 12 (9, 24) | 0.005 |
| 30-day re-admission | 4 (33.3) | 3 (42.9) | 1 (20.0) | 0.576* |
| AKI | 12 (13.5) | 7 (10.8) | 5 (20.8) | 0.217* |
| AKI staging   - 1 - 2 - 3 | 9 (75)  2 (16.7)  1 (8.3) | 6 (85.7)  1 (14.3)  0 | 3 (60.0)  1 (20.0)  1 (20.0) | 0.682* |

Abbreviations: AKI, acute kidney injury; BMI, body mass index; COPD, chronic obstructive pulmonary disease; CVA, cerebrovascular accident; Cr, creatinine; GFR, glomerular filtration rate; CMO, complex major operation; MAJ, major surgery; Physiological and Operative Severity Score for the enUmeration of Mortality and Morbidity, POSSUM; IBD, inflammatory bowel disease; ASA, american society of anaesthesiology; PCA, patient-controlled analgaesia; FTC, corrected flow time; DO2i, delivered oxygen; CRP, c-reactive protein; OIR, overnight intensive recovery

*Fisher’s exact test

**Supplementary table S4** **Clinical outcomes and relation to AKI categories and NGAL results**

|  | **No** | **Hospital LOS (days)** | **Re-admission (%)** | **Peri-operative complications (%)** | **1-year mortality (%)** | **8-year mortality (%)** |
| --- | --- | --- | --- | --- | --- | --- |
| pNGAL (T2 or T3) | 89 |  |  |  |  |  |
| - pNGAL-/Cr- - pNGAL+/Cr- - pNGAL-/Cr+ - pNGAL+/Cr+ | 7  70  3  9 | 12 (12, 15)  9 (6, 12)  14 (7, 24)  20 (10, 24) | 1 (14.3)  8 (11.4)  0  4 (44.4) | 6 (85.7)  37 (52.9)  3 (100)  9 (100) | 0  2 (2.9)  1 (33.3)  3 (33.3) | 1 (14.3)  18 (25.7)  1 (33.3)  4 (44.4) |
| p value |  | 0.052 | 0.079* | 0.005 | 0.005* | 0.525 |
| uNGAL (T2 or T3) | 89 |  |  |  |  |  |
| - uNGAL-/Cr- - uNGAL+/Cr- - uNGAL-/Cr+ - uNGAL+/Cr+ | 19  58  6  6 | 12 (8, 15)  8.5 (6, 12)  17 (7, 24)  17 (10, 24) | 3 (15.8)  6 (10.3)  2 (33.3)  2 (33.3) | 15 (79.0)  28 (48.3)  6 (100)  6 (100) | 1 (5.3)  1 (1.7)  1 (16.7)  3 (50.0) | 4 (21.1)  15 (25.9)  1 (16.7)  4 (66.7) |
| p value |  | 0.041 | 0.168* | 0.002 | 0.002* | 0.168 |

Abbreviations: pNGAL, plasma neutrophil gelatinase-associated lipocalin; uNGAL, urine neutrophil gelatinase-associated lipocalin; Cr, serum creatinine; LOS, length of stay

Plasma NGAL cutoff is 250 ng/mL and urine NGAL cutoff 100 ng/mL

**Supplementary figure S1 Receiver operating characteristics curve analysis of acute kidney injury prediction based on plasma neutrophil gelatinase-associated lipocalin at 3 timepoints**


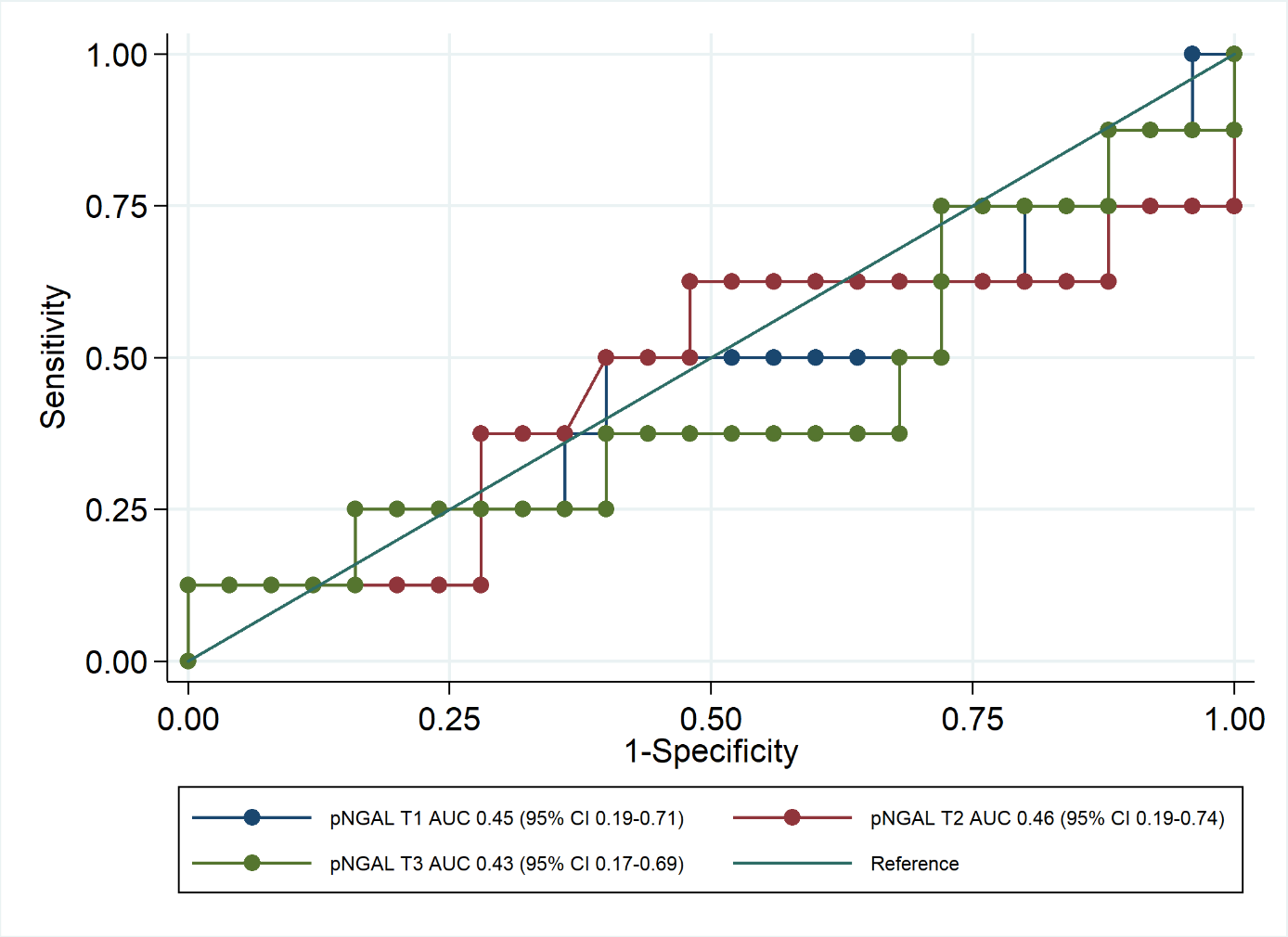


Abbreviations: pNGAL, plasma neutrophil gelatinase-associated lipocalin; uNGAL, urinary neutrophil gelatinase-associated lipocalin; AUC, area under the curve; CI, confidence interval

**Supplementary figure S2 Receiver operating characteristics curve analysis of acute kidney injury prediction based on urine neutrophil gelatinase-associated lipocalin at 3 timepoints**


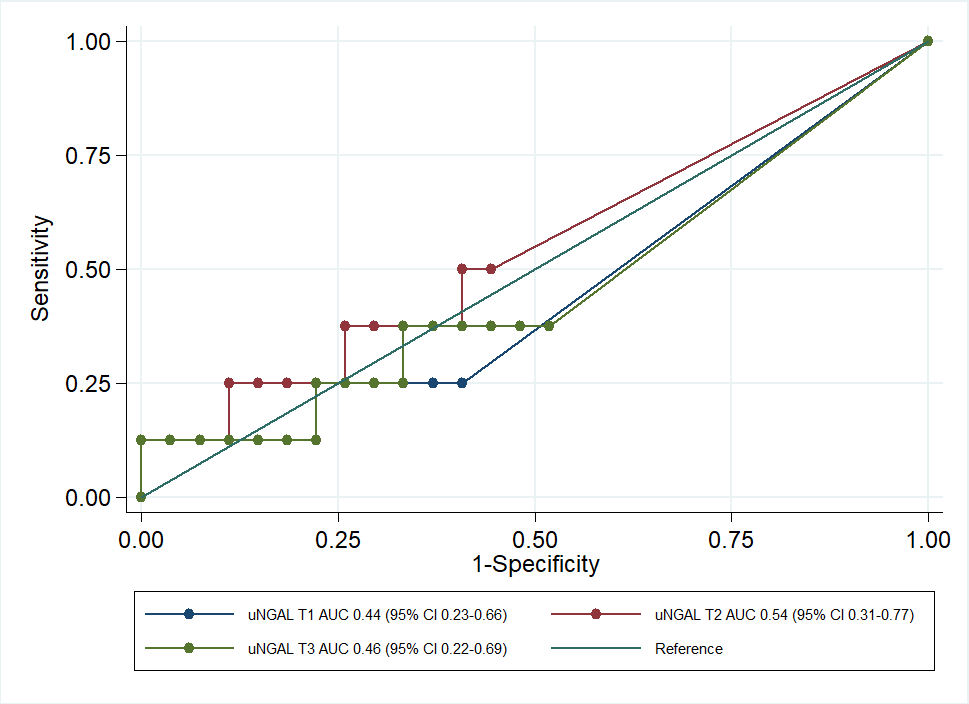


Abbreviations: pNGAL, plasma neutrophil gelatinase-associated lipocalin; uNGAL, urinary neutrophil gelatinase-associated lipocalin; AUC, area under the curve; CI, confidence interval

**Supplementary figure S3 Levels of baseline plasma and urine neutrophil gelatinase-associated lipocalin based on different stages of colorectal cancer**

| 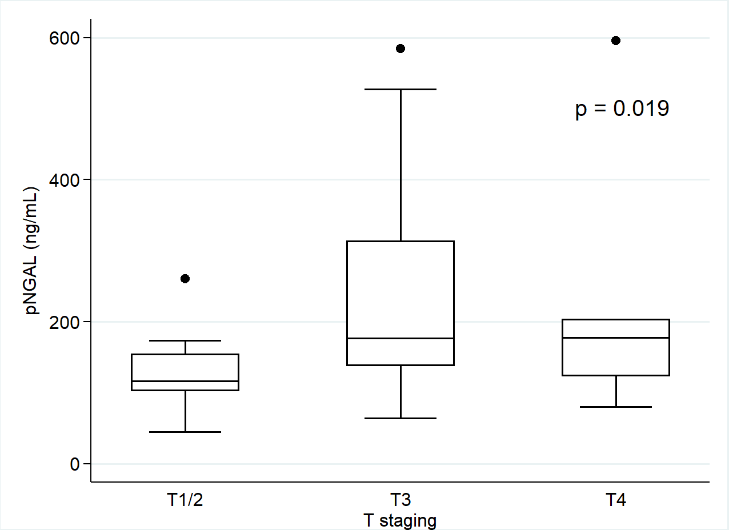 | 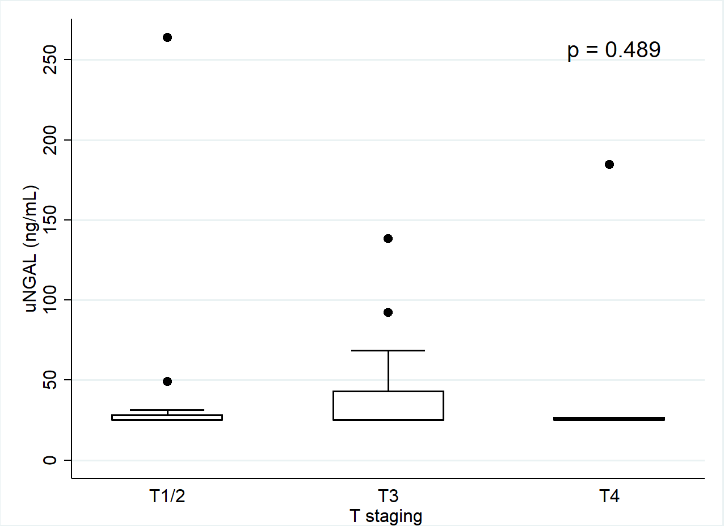 |
| --- | --- |
| 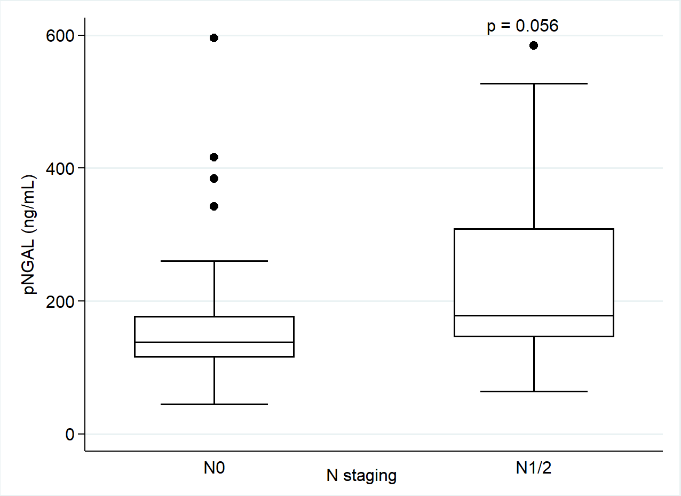 | 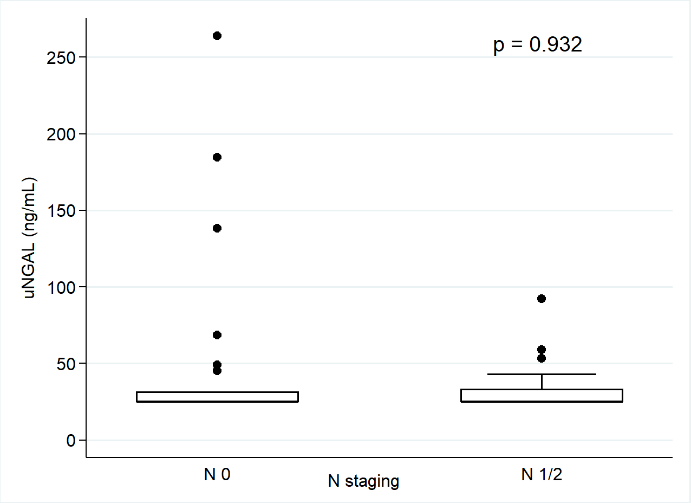 |
| 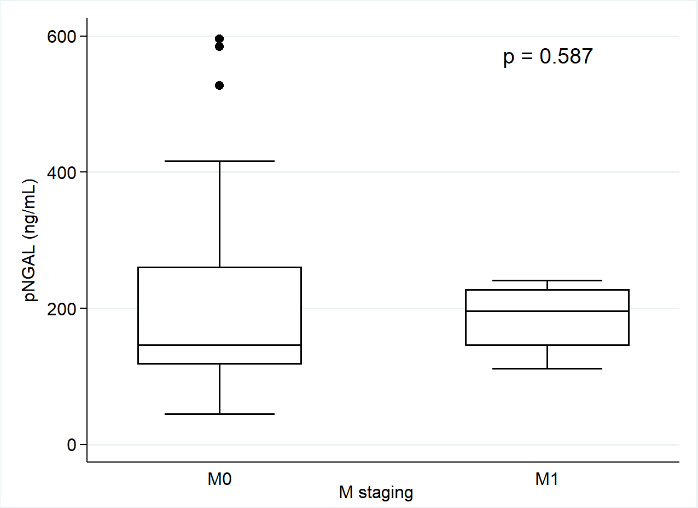 | 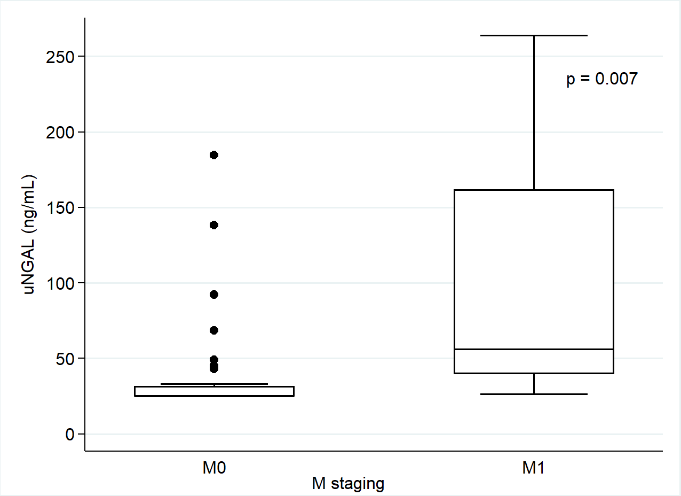 |

Abbreviations: pNGAL, plasma neutrophil gelatinase-associated lipocalin; uNGAL, urinary neutrophil gelatinase-associated lipocalin; M, metastatic disease; N, nodal disease

**Supplementary figure S4** **Spearman’s rank correlation between baseline plasma and urine neutrophil gelatinase-associated lipocalin**


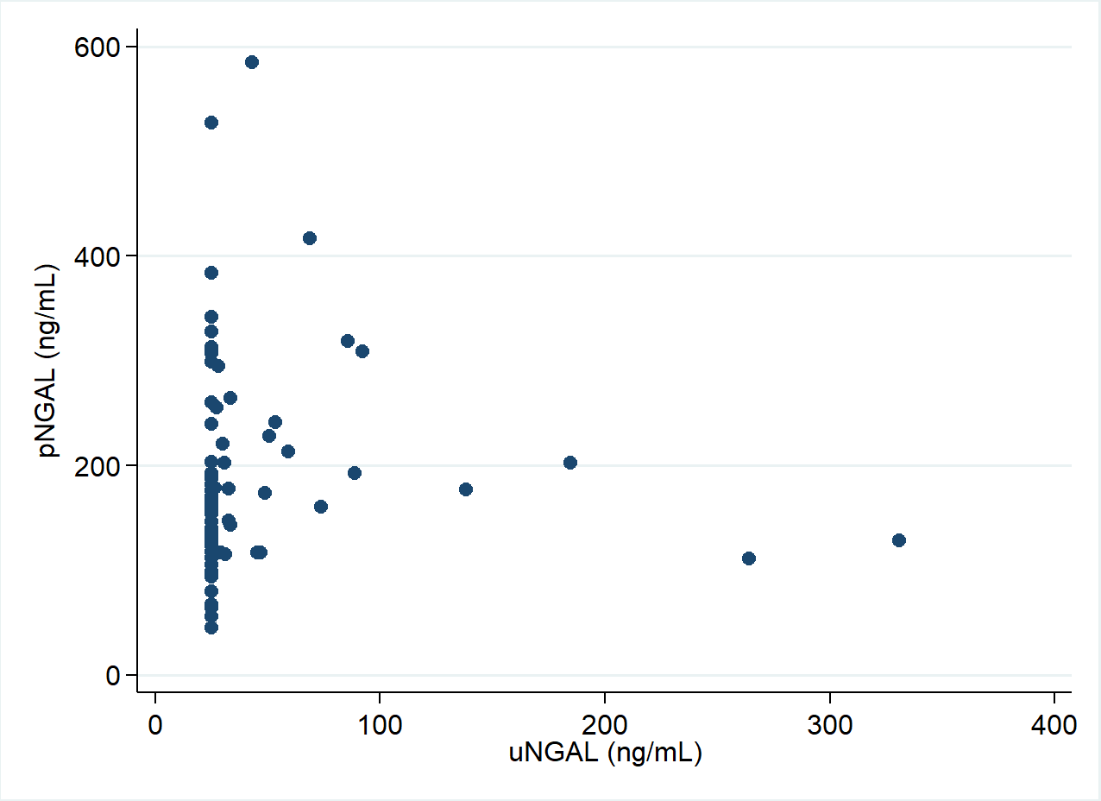


r = 0.30, p = 0.006

Abbreviations: pNGAL, plasma neutrophil gelatinase-associated lipocalin; uNGAL, urinary neutrophil gelatinase-associated lipocalin

**Supplementary figure S5 Eight-year survival curves by plasma neutrophil gelatinase-associated lipocalin and acute kidney injury categories**


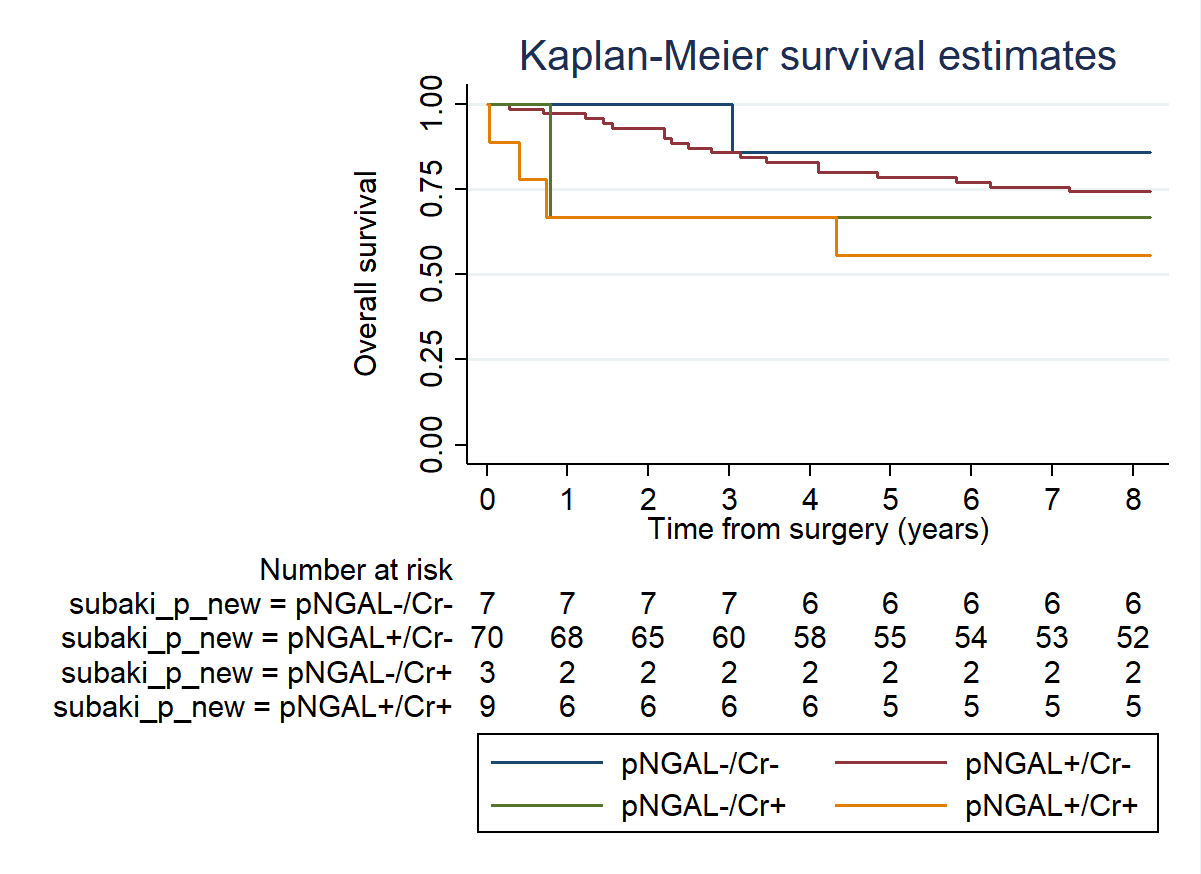


Abbreviations: pNGAL, plasma neutrophil gelatinase-associated lipocalin; uNGAL, urinary neutrophil gelatinase-associated lipocalin; Cr, serum creatinine

**Supplementary figure S6 Eight-year survival curves by urine neutrophil gelatinase-associated lipocalin and acute kidney injury categories**


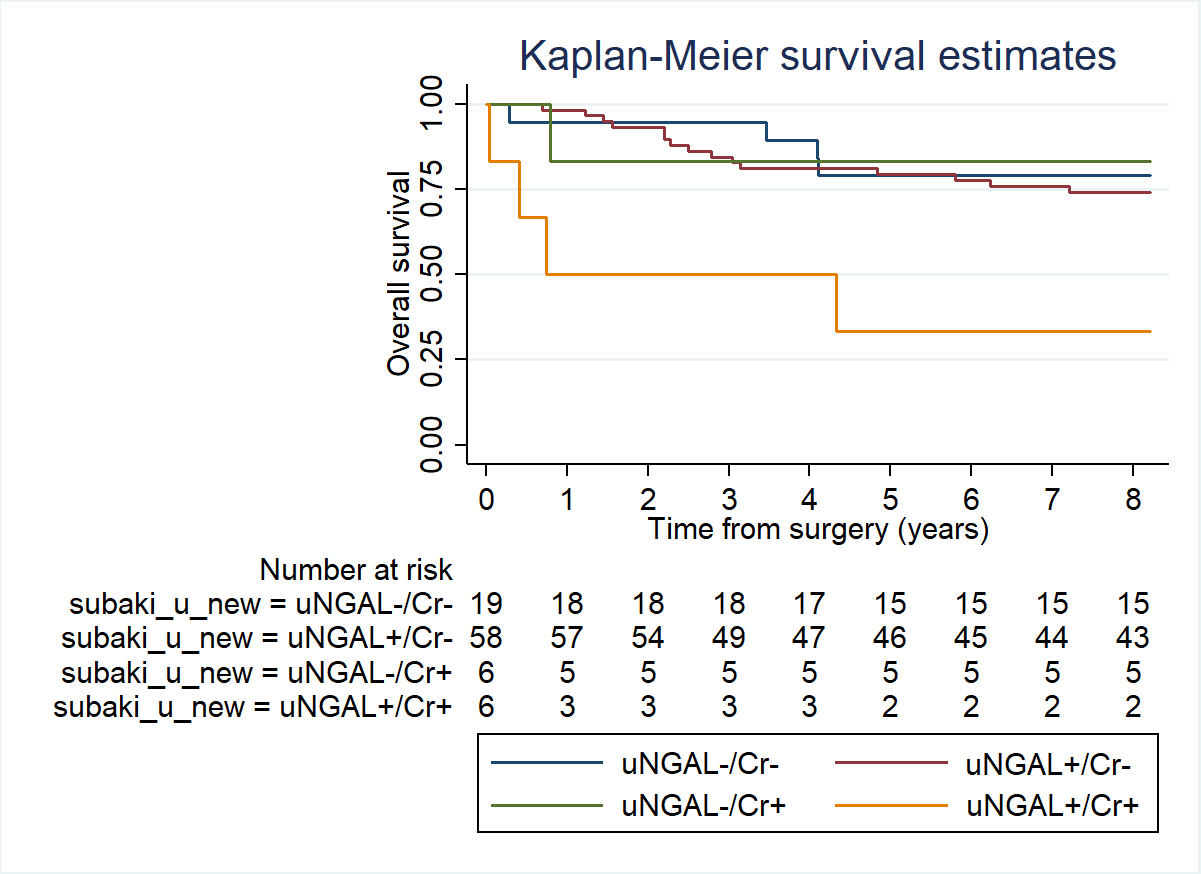


Abbreviations: pNGAL, plasma neutrophil gelatinase-associated lipocalin; uNGAL, urinary neutrophil gelatinase-associated lipocalin; Cr, serum creatinine
